# N-of-1 trials: The epitome of personalized medicine?

**DOI:** 10.1017/cts.2023.583

**Published:** 2023-07-03

**Authors:** Joyce P. Samuel, Susan H. Wootton, Jon E. Tyson

**Affiliations:** Department of Pediatrics, Center for Clinical Research and Evidence-Based Medicine, McGovern Medical School, The University of Texas Health Science Center at Houston, Houston, TX, USA

**Keywords:** Personalized medicine, precision medicine, randomized trials, n-of-1 trials

## Abstract

Observational studies are notoriously susceptible to bias, and parallel-group randomized trials are important to identify the best overall treatment for eligible patients. Yet, such trials can be expected to be a misleading indicator of the best treatment for some subgroups or individual patients. In selected circumstances, patients can be treated in n-of-1 trials to address the inherent heterogeneity of treatment response in clinical populations. Such trials help to accomplish the ultimate goal of all biomedical research, to optimize the care of individual patients.

## Introduction

The ultimate goal of clinical trials is to improve patient outcomes by better informing clinical practice and policy decisions. However, a growing number of critics note that many trials fail to accomplish this task [1,2]. The root of this challenge is multifactorial and often attributed to shortcomings in study design, result reporting, and limited generalizability. Here we present and discuss an underutilized approach, n-of-1 trials, that can provide tailored information to directly inform patient care [3].

Five conditions have been proposed that would increase the likelihood that a study will be informative in guiding clinical or policy decisions: (1) the study should address a clinically important and unresolved question; (2) a rigorous study design should be utilized that can provide an answer to the question; (3) enrollment goals and primary outcome completion should be feasible; (4) analysis should lend itself to valid interpretation; and (5) methods and results must be reported in a timely fashion [4]. Hutchinson and colleagues created surrogate measures for these conditions of informativeness and conducted a longitudinal cohort study, querying a clinical trials registry to quantify the proportion of randomized trials in specific disease areas that met these criteria [5]. Only 26% of 125 clinical trials met all conditions for informativeness. The authors suggested that improved scientific oversight might help address the methodologic weaknesses.

Even when the above criteria are met, obstacles remain in the direct application of trial results to patient care. Traditional parallel-group randomized trials generate estimates of average treatment effects among an often highly selected population. These results are then applied to individual patients who are judged to be sufficiently similar to the reference population in the study [6].

A problem with this practice is that universal administration of a therapy shown to be safe and effective on average may inadvertently harm some patients or be less beneficial than a generally less effective therapy. Subgroup analyses are often employed to identify which groups of patients may respond differently than other groups. However, subgroup analyses are often underpowered to identify a clinically meaningful difference between subgroups. Negative results may be misleading and require careful evaluation of their credibility [7,8].

## Personalized medicine: Shortcomings and an alternative approach

Personalized medicine has developed in response to the observation of significant variability in disease expression and treatment response across diseased individuals. The terms “personalized” or “precision” medicine are commonly used to refer to the application of pharmacogenomics to tailor treatment decisions based on an individual’s genetic profile. Leveraging our understanding of how specific genetic variants might affect the body’s response to a drug can, in theory, allow treatment choices to be personalized for the individual patient. Significant innovation in pharmacogenetics has been made in several clinical fields, including cardiovascular disease and oncology [9,10].

Despite major advances over the last two decades in human genome sequencing, mapping of interindividual genetic variations, and identification in gene-drug interactions, this field has yet to produce the widespread benefits and broad transformation of clinical medicine that have been predicted [11]. Beyond genetic factors, variation in treatment response is also influenced by unique physiological and biochemical characteristics as well as environmental exposures. This complex interplay leads to heterogenous treatment effects that generally will not be predictable based on any single clinical characteristic, genetic variation, or biomarker.

Under certain circumstances, this heterogeneity can be addressed in treating the individual patients using the therapy shown to be best for them in an n-of-1 clinical trial. When there is clinical equipoise on which treatment (or whether any treatment) is optimal for the individual patient, an n-of-1 clinical trial may be employed under certain conditions to determine the preferred treatment for that individual.

N-of-1 trials, also called “personalized trials,” employ a repeated crossover design in a single patient to allow direct comparison of treatment effects in the participant. Treatment order is preferably randomized and the repetition of treatment periods improves the ability to distinguish more precise differences in the estimates of treatment effects within the participant. Many design elements, including blinding, protocolized and objective outcome assessment, and washout periods between treatments, may be borrowed from traditional parallel group trials to minimize bias. Bayesian analyses may also be used to identify the probability of benefit, which is not directly assessed from conventional frequentist analyses [12]. As interventions are alternated, data are systematically collected on the individual’s response to the interventions, allowing for an unbiased comparison of treatment effects that can directly inform treatment decisions for the individual. When a series of these trials are conducted in similar patients, the results can be aggregated across the participants to produce population-level estimates of treatment effects [13].

The interested reader is referred to various examples of n-of-1 trials, including ours in treating children and adolescents with hypertension and other circumstances beyond assessing treatment effectiveness, modification of personal health behaviors, assessing behavioral interventions, and evaluating treatment harms [14–20]. The evidence base on n-of-1 trial methodology has grown rapidly in the last decade and addresses advanced issues in the design [21,22], analysis [23], and reporting quality [24,25].

## Indications for n-of-1 trials

N-of-1 trials are appropriate when there is clinical equipoise, and the comparative effectiveness of the treatment options is in doubt. This occurs commonly in clinical practice, particularly when well-performed comparative effectiveness studies are not feasible (as for rare diseases), or their results have limited generalizability. N-of-1 trials may be particularly important when the treatment risks and benefits are most uncertain, as for populations that are often excluded from large randomized trials, such as vulnerable populations (ex. children and the elderly) or those with complicated comorbidities or rare diseases. For these patients, trial data may not be available, and decisions would otherwise be based on biological plausibility, observational studies, or anecdotal experience.

N-of-1 trials are most worthwhile for chronic conditions, which will require long-term maintenance therapy. Examples of previous use cases include hypertension, attention-deficit hyperactivity disorder, chronic pain, osteoarthritis, and atrial fibrillation [26,27]. A self-limited disease or one with infrequent clinical manifestations would not be appropriate for this study design. Participation in n-of-1 trials has the potential to promote self-efficacy and health literacy, especially when they are used to evaluate health behaviors including physical activity, stress reduction, and dietary changes. Additionally, by directly involving patients in the decision-making process, adherence to the selected treatment may be improved. Examples of various uses of n-of-1 trials are shown in the Table 1.

**Table 1.** Examples of n-of-1 trials for various use cases

	Clinical question	Design features	Treatment periods	Use of washout	Analytic approach	Digital tools	Outcome assessment
Pharmacologic treatment effectiveness	Evaluate individual responses to stimulants among children with attention-deficit/hyperactivity disorder [35]	Double-blind, placebo-controlled design: stimulant vs placebo or alternative stimulant	One week per condition, 2 conditions tested (each 3x) over total 6 weeks	No washout periods	None described Responder status categorized based on symptom scores	None	Parent, teacher, and self-rating of ADHD symptoms: assessed during and at the end of each treatment period
Assess harms	Verify perceived side effects of statin tablets [36]	Double-blind, placebo-controlled design: statin vs placebo vs no tablet	One month per condition, 3 conditions tested (each 4x) over total 12 months	No washout periods	Mixed (multilevel) linear models, multiple imputation for missing data	Smartphone application to collect daily symptom scores	Self-report of side effects: assessed daily
Rare disease	Assess effects of dietary cholesterol supplementation on behavior among children with Smith-Lemli-Opitz syndrome [37]	Double-blind, placebo-controlled design: liquid egg yolks vs egg substitute (placebo)	Two weeks per condition, 2 conditions tested (each 1x) over total 10 weeks including washout periods	2-week washout with baseline cholesterol therapy between each treatment period	Paired *t*-test	None	Parent report of hyperactivity: assessed at the end of each 2-week treatment period
Nonpharmacological intervention	Assess the effect of bright white light therapy for depressive symptoms in cancer survivors [38]	Single-blind, sham-controlled design: bright white light vs dim red (sham)	Three weeks per condition, 2 conditions tested (each 2x) over total 12 weeks	No washout periods	Autoregressive model: treatment arm as main exposure, adjusted for time and accounting for autocorrelations of the order 1	Smartphone application to collect daily symptom scores, aggregate data by treatment period, and deliver trial results to participants	Self-reported pain, physical performance score, body weight, step count: assessed daily
Lifestyle changes	Evaluate presumed patient-selected triggers for atrial fibrillation [15]	No blinding Six 1-week periods of trigger exposure or non-exposure. Trigger examples: alcohol, caffeine, lack of sleep	One week per condition, 2 conditions tested (each 3x) over total 6 weeks	No washout periods	Bayesian generalized linear model	Smartphone application to deliver reminders on triggers, collect daily AF self-report, and deliver trial results to participants Mobile electrocardiogram device which paired with smartphone	Self-report of AF episode assessed daily, instructed to use mobile ECG device to record when they suspected an abnormal rhythm

The approach requires a patient who is eager to collaborate in an n-of-1 trial. As most patients will be unfamiliar with the concept, caregivers should be forthcoming about the notion of therapeutic uncertainty, and should review the risks and benefits of rotating between treatment options to repeatedly assess treatment response. Patient perspectives on participation may be influenced by cultural factors, and understanding these may help improve health equity in n-of-1 trials. Some barriers to participation identified in qualitative studies among racial and ethnic minority patients include mistrust in healthcare systems, language barriers, and a reluctance to undergo blinded treatment [28].

This trial design is most feasible when the treatment effects have a rapid onset and offset of action. As in traditional crossover trials, carryover effects must be considered, and are more likely to occur when the outcome is assessed before the effects of the previous treatment have sufficiently diminished. This can be addressed with the use of washout periods between treatments. The treatment periods should be long enough to capture a difference in the outcome, but short enough to be practical for the clinician and the patient. The number of times a given treatment is repeated will affect the precision (or power) in differentiating the relative effects of each treatment. Finally, this approach requires the use of measurable and clinically relevant treatment targets. The measurement chosen to assess treatment effects should not only be valid and reliable but also convenient enough that patients would be willing to undergo the assessment repeatedly.

## Improving the usefulness of n-of-1 trials

N-of-1 trials are not immune to many of the same issues that plague some randomized trials and limit their informativeness to clinical practice. If the study designs are susceptible to bias or the results are poorly reported, their usefulness to all stakeholders, including patients, will be hindered. The Consolidated Standards of Reporting Trials extension for n-of-1 trials provides guidance on the preparation and appraisal of reports of n-of-1 trials in the medical literature [24]. Trial design should be reported explicitly, including the planned number of periods, the duration of each period, whether a run-in or washout period is used, and whether the design was individualized to each participant. The outcome measurement tools should be comprehensively described, including measurement properties such as validity and reliability. If the order of treatments is randomized, the method used to generate the allocation sequence should be described. The statistical analysis should include a description of how carryover effects, period effects, and intra-subject correlation are handled. Changes from the original plan for treatment periods, including the number and sequence of periods completed, should be explained. Comprehensive reporting on the design and execution of n-of-1 trials will improve their applicability to clinicians who are interested in employing these trials to inform the care of their patients.

## Increasing uptake

Several barriers to widespread implementation in clinical practice should be addressed, including the doubt among clinicians and patients about whether the costs and burdens of n-of-1 trials outweigh the benefits. Very few randomized trials have been conducted to assess whether n-of-1 trials, when compared to usual care, produce improved clinical outcomes [27]. While most studies were equivocal for the primary outcome, several studies identified significant differences in secondary outcomes, including satisfaction with care and increased shared decision-making. However, most of the studies did not reach their predefined sample size targets, and insufficient sample sizes may have reduced their power to identify the benefit of n-of-1 trials.

## Digitizing n-of-1 trials

Another major constraint to the widespread application of this approach in routine clinical practice is the lack of tools and infrastructure that would allow a clinician to conduct n-of-1 trials in a busy clinical setting. For this reason, most n-of-1 trial activity has been limited to funded clinical research conducted in academic settings. The momentum to incorporate this approach into everyday clinical practice is usually stalled by loss of research funding or diminished interest among clinicians without a research background. Open-access digital platforms and mobile applications to support the design, conduct, and analysis of n-of-1 trials would reduce logistical barriers and might increase use. Integration of such digital platforms into the electronic health record would facilitate the implementation of n-of-1 trials into clinical practice. Additionally, online platforms would promote sharing of resources and knowledge, allowing protocols to be utilized across multiple institutions and populations. N-of-1 trial apps allow patients to self-track symptoms and may also integrate with other mobile health technologies to allow for passive outcome data collection. Further development of these technologies is critical to advancing the integration of n-of-1 trials into clinical practice [29–31].

## Ethical and regulatory oversight

Additional considerations in the implementation of n-of-1 trials include the issue of the ethical and regulatory oversight requirements. Currently, there are no national guidelines that address how institutional review boards should approach consent for n-of-1 trials, but some consensus is forming [32,33]. As we have previously outlined [34], if certain conditions are met, a less formal consent process could be sufficient. These criteria include 1) a patient’s well-being remains the primary motivation, 2) there is no incremental increase in risk relative to usual care, and 3) the patient understands the process and agrees to participate. Some n-of-1 trials may qualify as quality improvement whereas others may only require a streamlined consent approach (verbal discussion accompanied by an information sheet without the need for signature).

## Conclusions

In the era of precision medicine, n-of-1 trials offer a pathway to provide patient-centered care based on evidence that is generated directly from the individual patient. Embedding these trials into clinical practice may improve patient outcomes and reduce wasted time and resources on therapies that don't work best for the patient. Given the current emphasis on customized, patient-centered care, the n-of-1 trial approach has the potential to be the epitome of personalized medicine.
